# RGD-modified alginate enhances viability, metabolic reprogramming, and cytokine secretion profiles in encapsulated mesenchymal stromal cells

**DOI:** 10.1038/s41598-026-42864-7

**Published:** 2026-05-13

**Authors:** Kadriye Güven, Anil Dhawan, Celine Filippi

**Affiliations:** 1 DhawanLab, Roger Williams Institute of Liver Studies, King’s College London, King’s College Hospital, London, SE5 9RS UK; 2 Paediatric Liver, GI and Nutrition Centre and MowatLabs, King’s College London, King’s College Hospital, London, UK

**Keywords:** Mesenchymal stromal cells, Umbilical cord-derived MSCs (UC-MSCs), Alginate microencapsulation, Mitochondrial biogenesis, HIF1A, Cell therapy, RGD-modified biomaterials, Biotechnology, Cell biology, Stem cells

## Abstract

Mesenchymal stromal cells (MSCs) have been widely studied for their regenerative and immunomodulatory properties. However, clinical translation is hindered by a lack of, or limited, in vivo retention due to immune-mediated clearance. Biomaterial-based encapsulation, particularly using alginate hydrogels, offers a promising strategy to enhance MSC persistence and functionality. This study aimed to evaluate the impact of unmodified and RGD-functionalized GMP-grade alginate matrices on umbilical cord-derived MSCs (UC-MSCs) viability, mitochondrial function, and cytokine secretion profile. Cryopreserved UC-MSCs were encapsulated in GMP-compatible ultrapure alginates, an unmodified alginate (SLG20) and (G-RGD) modified with Arg-Gly-Asp (RGD) peptides, and compared to 2D cultures over five days for viability, metabolic activity, cytokine secretion, and mitochondrial function. MSCs encapsulation in G-RGD alginate significantly enhanced viability and modified cytoskeletal organisation compared to SLG20. While encapsulation resulted in 58% reduction in OXPHOS, relative to 2D culture at Day 0 (*p* < 0.01), with no significant difference between SLG20 and G-RGD. G-RGD-encapsulated cells maintained significantly higher basal, ATP-linked, and maximal respiration (*p* < 0.01) than SLG20-encapsulated cells for up to five days. Notably, encapsulation triggered a 60-fold upregulation of *PGC1A* and a twofold increase in *HIF1A* expression by Day 3, indicating metabolic adaptation and mitochondrial biogenesis signalling. Early cytokine profiling showed that encapsulation increased VEGF secretion by approximately 11-fold compared to 2D MSCs. IL-6 secretion was 34.5% higher in G-RGD than in SLG20 at Day 1 and was markedly higher in encapsulated MSCs than in 2D cultures. Although TNF-α secretion remained low overall, levels were 49% higher in G-RGD than in SLG20 at Day 5. IL-10 levels were similar between matrices. Encapsulation reduced glycolytic output by 67% compared to 2D cultures and lowered *THY1 (CD90)* expression over time. This work was translation-focused, testing whether established RGD-related benefits are preserved under GMP-grade materials and can be clinically deployable with cryopreserved cells. Our findings reveal that a simple encapsulation in alginate microbeads, within 500 μm beads, creates a hypoxic environment for MSCs, similar to their natural niche, which strongly alters their functions as compared to the classical 2D plastic culture. G-RGD alginate provided modest but consistent advantages over unmodified SLG20 in maintaining mitochondrial function and modulating cytokine secretion. Matrix composition remains a critical factor in shaping MSC behaviour, and G-RGD ultrapure alginate represents a promising material for optimising cell-based therapies under clinically relevant conditions.

## Introduction

Mesenchymal stromal cells (MSCs) have attracted particular attention in the field of regenerative medicine due to their promotion of tissue repair, their potent immunomodulatory properties, combined with their minimal ethical issues^1–3^. Their accessibility and ease of expansion have enabled widespread basic and clinical investigations across a broad range of diseases, from inflammatory disorders to acute organ failure. However, despite encouraging preclinical data, clinical efficacy remains limited due to rapid immune clearance, resulting in poor target tissue retention and short-lived therapeutic effect^4–6^.

To address this, biomaterial-assisted delivery systems have emerged as a strategy to enhance MSC survival, persistence, and functionality. Among these, alginate hydrogels have emerged as clinically usable matrices owing to their biocompatibility, inertness, and adaptable or modifiable mechanical properties^7–10^. GMP-grade alginates have now been developed to align with regulatory requirements for translational applications. GMP-grade alginate was selected to ensure translational relevance and alignment with regulatory frameworks for advanced therapy medicinal products (ATMPs). Compared to research-grade materials, GMP-grade alginates offer (i) low endotoxin content and high purity, reducing the risk of unintended immune activation, (ii) improved batch-to-batch consistency, including certificate-backed documentation of RGD substitution, and (iii) traceability and quality documentation required for clinical product development. Alginate-based encapsulation offers physical immuno-protection by forming a semi-permeable barrier that enables the diffusion of nutrients and paracrine factors while preventing immune cell attack^11^. Although MSCs were once considered hypoimmunogenic, they are now recognised as immune-evasive but still susceptible to clearance by host NK and T cells^12^. By physically shielding transplanted MSCs from this immune pressure, alginate encapsulation can prolong their persistence and enable sustained paracrine activity essential for repair. MSC functions also rely on appropriate cell–matrix interactions that maintain their phenotype and therapeutic potency. Functionalisation of alginate with Arg-Gly-Asp (RGD) peptides enables integrin-mediated adhesion, better recapitulating the extracellular matrix environment and supporting MSC survival signalling^13–15^.

At the same time, advances in MSC biology highlight the central role of metabolic programming in dictating therapeutic function. Native MSC niches, such as bone marrow or perivascular tissues, are typically hypoxic and mechanically dynamic, favouring glycolysis over oxidative phosphorylation (OXPHOS)^16,17^. In contrast, atmospheric 2D culture disrupts this balance, leading to a metabolic drift and functional modifications. The biophysical properties of the encapsulating matrix and cell–matrix interactions significantly impact MSC bioenergetics and therapeutic output. MSCs exhibit a high degree of metabolic plasticity, relying on either glycolysis or OXPHOS depending on their environmental conditions^18,19^. Under reduced oxygen tension, hypoxia-inducible factor 1-alpha (*HIF1A*) is stabilised, promoting a metabolic shift towards glycolysis to support cell survival^20,21^. In MSCs, such hypoxic adaptation has been shown to preserve their stemness and enhance their paracrine function^21–23^. Mitochondrial biogenesis, regulated by *PGC1A*, *NRF1/2*, and *TFAM*, plays a central role in maintaining mitochondrial integrity and energy production during metabolic adaptation^24^. However, its activation under hypoxia is context-dependent, mitochondrial biogenesis being suppressed or transiently induced depending on the intensity and duration of hypoxic exposure^25^.

We and other groups have showed that MSC can support the function of a variety of cells when co-encapsulated^26^. However, we never focused on the specific response of MSCs to the encapsulation process. Because our group focuses on the development of new therapies, requiring adherence to GMP regulations, this study aimed at comparing the behaviour of MSCs - isolated, cultured and cryopreserved in a GMP-like fashion - in 2 GMP-compliant alginates: SLG20, an ultrapure sterile alginate, and an RGD-modified alginate (G-RGD). We specifically looked at the ability of the hydrogels to support umbilical cord-derived MSC (UC-MSC) viability, bioenergetics and cytokine secretion. While RGD‑modified alginate matrices have been widely explored in experimental MSC culture systems, most prior studies employ various types of research‑grade polymers, freshly harvested cells, or two‑dimensional and spheroid‑based formats. In contrast, the present study adopts a clinically constrained framework, integrating GMP‑grade alginates (SLG20 vs. G‑RGD), cryopreserved UC‑MSCs reflecting real‑world clinical workflows, and microbead formulations compatible with intraperitoneal delivery.

## Materials and methods

### Culture and cryopreservation of UC-MSCs

All procedures involving human-derived biological materials were carried out in accordance with relevant institutional and national guidelines and regulations. The study was approved by the King’s College Hospital Research Ethics Committee (Reference: LREC 01–016). Human umbilical cord–derived mesenchymal stromal cells were obtained from the Anthony Nolan Trust (UK) with written informed consent from all donors or their legal guardians, in accordance with the Declaration of Helsinki. Human umbilical cord–derived mesenchymal stromal cells (UC-MSCs procured from the Anthony Nolan Trust, UK) were isolated from Wharton’s jelly as previously described^26^ and cryopreserved at temperatures lower than – 130 °C. UC-MSC vials were thawed and expanded for experimental use in α-Minimal Essential Medium (α-MEM; Thermo Fisher Scientific, UK cat# 32561094) supplemented with 5% Stemulate^®^ pooled human platelet lysate (Cook Regentec, USA, Ref# G35220), 1% penicillin‐streptomycin (P/S; (cat# 15140122)), and 1 mM sodium pyruvate (Lonza, Switzerland, cat# 13‐115E). Cells were maintained at 37 °C in a humidified 5% CO₂ incubator. After reaching 70–80% confluence within a week, the cells were passaged and expanded, with an approximate density of 3000 cells per cm^2^. Passage 4 (P4) cells were used for all experiments to ensure optimal viability and functionality. For cryopreservation, cells were resuspended in α-MEM supplemented with 10% DMSO (Sigma‐Aldrich, UK, cat# D2650) and 5% human serum albumin (Zenalb 20, UK, PL 08801/0007) at a density of 1 × 10⁶ cells/mL and stored at − 140 °C. All reagents were obtained from Thermo Fisher Scientific, UK, unless otherwise specified.

### Encapsulation of cells in alginate hydrogels

Cryopreserved UC-MSCs were thawed and encapsulated in alginate microbeads using a B-395 Pro encapsulator (Buchi Labortechnik AG, Flawil, Switzerland), while the IE-50R encapsulator (Inotech Encapsulation AG, Dottikon, Switzerland) was used in early optimisation steps, as previously described^27^. Click or tap here to enter text.Before encapsulation, ultrapure sodium alginate with low viscosity and high guluronate (PRONOVA™ SLG20; NovaMatrix, Sandvika, Norway) was dissolved in 0.9% (w/v) NaCl to reach a final concentration of 1.5% (w/v). For RGD-modified alginate, lyophilised NOVATACH™ MVG GRGDSP alginate (NovaMatrix, Sandvika, Norway) was used. This GMP-grade alginate is based on high-G sodium alginate covalently coupled to the GRGDSP peptide (Gly-Arg-Gly-Asp-Ser-Pro) via stable amide linkage, enabling integrin-specific binding that mimics natural extracellular matrix interactions. The degree of RGD peptide substitution is indicated in the manufacturer’s certificates of analysis for each batch. For batch BP-2109-22, the degree of substitution was 0.375%, and for batch BP-2311-20, the substitution degree was 0.382%. Both batches were used in this study. The alginate was reconstituted in 0.9% (w/v) NaCl to achieve a final concentration of 1.1%. This lower concentration was selected based on the manufacturer’s (NovaMatrix) recommendation to optimise viscosity and droplet formation for GRGDSP-functionalised alginate, as RGD coupling alters gelation properties. The adjustment ensured comparable bead size and structural integrity to 1.5% unmodified SLG20, in line with previous encapsulation reports^26^. The mixture was gently combined with the cells at a density of 0.8 × 10⁶ cells/ml of alginate, corresponding to approximately 50–55 cells per 500 μm bead at day 0, based on bead volume (~ 65 nL). The suspension was carefully prepared to avoid bubble formation and subsequently filtered through a 100 μm mesh to ensure uniformity. Encapsulation was performed using a regularly maintained Buchi B-395 Pro encapsulator, which consistently produced highly uniform bead suspensions. Microbeads were generated using a 200 μm nozzle, cross-linked in a 100 mM CaCl₂ (1.2%w/v) solution for 10 min and washed twice with 0.9% (w/v) NaCl. Encapsulation efficiency was not directly quantified; however, the cells were premixed with alginate prior to extrusion, ensuring that the vast majority of cells were incorporated into microbeads during droplet formation, with free single cells rarely observed in the CaCl₂ crosslinking bath. Vibrational extrusion systems such as the Buchi B-395 Pro encapsulator have been reported to achieve encapsulation efficiencies exceeding 90–95% under optimised conditions^28^. For cell culture, the microbeads were maintained in Williams E medium (cat# A1217601) supplemented with 10% (v/v) heat‐inactivated foetal calf serum (Gibco, cat# 10270106), 2 mM L‐glutamine (cat# 25030081), 10 mM HEPES (cat# 15630056), 10 mg/L insulin (Sigma-Aldrich, UK, cat# I9278), 5.5 mg/L transferrin (Sigma-Aldrich, UK, cat# T8158), 670 µg/L sodium selenite (ITS-G supplement, cat# 41400045), 10⁻⁷ M dexamethasone (cat# D4902), 100 U/mL penicillin, and 100 µg/mL streptomycin (P/S, cat# 15140122). One millilitre of microbeads was cultured in 4 mL of complete medium per well of a 6‐well plate in a humidified incubator at 37 °C with 5% CO₂ for up to 5 days, with medium replacement every 2–3 days. Images of the microbeads were captured using an EVOS FL Auto 2 microscope (Thermo Fisher Scientific, UK), and an inverted microscope (DMi8, Leica Microsystems Ltd.) was used for higher-magnification imaging, including Z-stack acquisition for 3D assessment of encapsulated cells.

### Microbead size measurement

Microbead size was quantified using the Fiji (ImageJ 2.16.0/1.54 g) software, and the bead size distribution was compared between UC-MSCs encapsulated in SLG20 and G-RGD alginate formulations. Beads were imaged under consistent magnification, and diameter measurements were performed. All microbeads were generated using a GMP-compliant encapsulation process, and their diameters met predefined clinical release criteria, ensuring suitability for translational application.

### Cell viability assessment

A small volume of beads (100–200 µl) was placed in a 3 mL petri dish and gently washed with calcium- and magnesium-free DPBS (Gibco, cat# 14190144). FDA (10 µg/mL, Sigma #F7378, UK) staining solution was prepared from concentrated stocks and combined as specified. The beads were incubated with the staining solution for 15 min at 37 °C, washed three times with DPBS, and then imaged to assess cell viability.

### Phalloidin staining in alginate beads

Alginate beads (500 µl) were transferred into 35 mm petri dishes using a wide-bore pipette and fixed in 4% methanol-free formaldehyde (PFA) (Pierce™ 16% Formaldehyde (w/v), Methanol-free Cat# 28906) in DPBS for 20 min at RT with gentle shaking. The beads were then washed three times with DPBS before permeabilization of the cells in 0.1% Triton X-100 in DPBS for 10 min at RT. Following a further three washes with DPBS by shaking, the microbeads were incubated in the dark with phalloidin CF568 conjugate (Biotium, cat# 00044-T), prepared at a 1:40 dilution by mixing 5 µL of stock with 200 µL DPBS per well of a 24-well plate. Staining was performed for 60 min at room temperature (RT). Subsequently, the beads were rinsed in DPBS for 5 min. Finally, the beads were transferred to glass-bottom dishes containing a 50% glycerol solution to minimise movement during imaging using a fluorescence microscope.

### MTT assay for viability in alginate beads

A 5 mg/mL MTT solution was prepared in calcium- and magnesium-free DPBS, using MTT powder (Sigma-Aldrich, UK, cat# D8537). The solution was thoroughly vortexed, filter-sterilised through a 0.2 μm syringe filter, aliquoted, and stored at − 20 °C until use. Alginate beads containing UC-MSCs (100 µL packed volume including medium) were transferred into a 24-well plate (three wells per condition) and gently rinsed with calcium- and magnesium-free DPBS (Gibco, cat# 14190144) to remove residual culture medium prior to the MTT assay. Fifty microlitres of stock MTT solution was added to each well (final concentration 0.45 mg/ml in 500 µl serum-free Williams’ E medium), and the plate was incubated at 37 °C/5% CO₂ for 4 h, allowing purple formazan crystals to form within the beads. The medium was carefully removed, and 0.25 mL DMSO was added to each well to dissolve the formazan, with the plate then shaken for 30 min. The solubilised formazan was transferred to a 96-well plate, and absorbance at 550 nm was measured using the FLUOstar^®^ Omega microplate reader (BMG LABTECH, Germany).

### Oxidative phosphorylation analysis

Encapsulated MSCs (0.5 × 10^6^ cells) were assessed for oxidative phosphorylation (OXPHOS) using an Oxygraph-2k high-resolution respirometer (Oroboros Instruments, Innsbruck, Austria). The two chambers (2 mL each) were filled with Williams E culture medium at 37 °C under gentle agitation (750 rpm). Oxygen consumption was measured at baseline, after adding 2.5 µM oligomycin (leak state), and following successive titrations of 1 µM CCCP (uncoupled state). Non-mitochondrial respiration was assessed after the addition of 2.5 µM antimycin A. Basal respiration, maximal respiratory capacity, reserve capacity, ATP-linked respiration, and the respiratory control ratio were determined. MSCs were either in cell suspension from 2D culture or encapsulated, with freshly thawed MSCs serving as controls.

### Fluorescence microscopy-based hypoxia measurements in microbeads

Microbeads were cultured in 6-well plates for up to 5 days at 37 °C under standard CO₂ incubation. The microbeads were stained with 5 µM Image-iT™ Green Hypoxia Reagent (Invitrogen™), with sodium dithionite at 1.5 mM concentration (Sigma-Aldrich, cat# 7775-14-6) serving as a positive control for mimicking hypoxic conditions. After 4 h of incubation, the beads were imaged by fluorescent microscopy.

### Depolymerisation of alginate capsules

The alginate beads were settled to remove the medium and resuspended in a solution containing a calcium chelator (0.2 M sodium citrate, 0.1 M EDTA, 10 mM HEPES, 0.1% glucose) on ice for 10 min to break down the alginate scaffold. The digestion solution was filtered through a 70-µm cell strainer to remove any remaining cross-linked alginate, followed by centrifugation at 300 g for 5 min to collect the released cells.

### RNA isolation and one-step RT- qPCR

At the desired time point, the culture medium was removed, and microbeads were washed twice with DPBS. The encapsulated cells were then retrieved by depolymerisation. Total RNA was then isolated using the Qiagen RNeasy Mini Kit (cat# 74104) following the manufacturer’s guidelines. The concentration and purity of the extracted RNA were determined using a Nanodrop spectrophotometer, and samples were stored at − 80 °C until further use. The mRNA levels of the target genes were quantified using the SuperScript™ III Platinum™ One-Step qRT-PCR Kit (Thermo Fisher Scientific, Life Technologies) according to the manufacturer’s instructions and run on an ABI Quant Studio 5384. Reactions utilised a 4 ng RNA template, with gene expression levels normalised to the housekeeping genes β-actin, GAPDH, and B2M at the specified time points. Reference gene stability was assessed across all experimental conditions, and β-actin, GAPDH, and B2M showed minimal Ct variability (coefficient of variation ≤ ~ 3%); therefore, the mean expression of these three genes was used for normalisation. The mRNA expression fold change relative to controls was calculated using the ΔΔCt method. The TaqMan^®^ probes (Thermo Fisher Scientific, USA) employed for quantitative PCR are listed as follows: *HIF1A* (Hs00153153_m1), *PGC1A* (Hs00173304_m1), *NRF1* (Hs00602161_m1), *NRF2* (Hs00975961_g1), *TFAM* (Hs00273372_s1), *TOMM40* (Hs01587378_m1), *COX4I1* (Hs00971639_m1); *CD90 (THY1)* (Hs06633377_s1) *GAPDH* (Hs02786624_g1), *B2M* (Hs00187842_m1), *ACTB* (β-actin; Hs01060665_g1).

### Mitochondrial membrane potential assessment via JC-1 staining (2D and 3D encapsulated cultures)

The cell mitochondrial membrane potential (ΔΨm) was assessed using the potentiometric dye JC-1 following the manufacturer’s instructions. (Thermo Fisher Scientific, Cat#T3168). Briefly, encapsulated MSCs and 2D-cultured MSCs were incubated with 5 µM JC-1 dye (Thermo Fisher Scientific) in serum-free WE medium at 37 °C with 5% CO₂ for 30 min. Following incubation, microbeads were washed twice with DPBS to remove excess dye prior to fluorescence analysis. Z-stack confocal images were acquired and subsequently subjected to maximum intensity projection. A threshold was applied to remove background fluorescence, and the mean fluorescence intensity within individually defined bead regions (ROIs) was quantified using the FIJI software (https://fiji.sc/).

### Enzyme-linked immunosorbent assays (ELISA)

The conditioned medium was collected and centrifuged at 300 g for 5 min, then immediately frozen in liquid nitrogen and stored at − 80 °C for subsequent analysis. The concentrations of Human IL-6, VEGF, IL-10, and TNF-α were measured using DuoSet ELISA kits (R&D Systems, USA): IL-6 (cat# DY206-05), VEGF (cat# DY293B), IL-10 (cat# DY217B), and TNF-α (cat# DY210-05), following the manufacturer’s instructions.

### Measurement of lactate and pyruvate in cell culture

Following microbead cell culture, conditioned media was collected and stored at − 80 °C until further analysis. Lactate was quantified using an NADH/NAD^+^-independent enzymatic assay based on lactate dehydrogenase (LDH) activity, as described by Bergmeyer (1963). A reaction solution (pH 9) containing 0.75 mM NAD^+^, 0.4 M hydrazine hydrate, and 0.4 mM glycine was prepared. For each sample, 20 µL was added to a Greiner F-bottom 96-well plate, followed by 180 µL of reaction buffer with LDH (calculated to deliver 5 IU per 2 mL reaction volume from a 550 IU/mL stock). Absorbance was measured at 572 nm, and lactate concentrations were determined via a standard curve generated with sodium DL-Lactate (Cat# L9700-100mL). Pyruvate was measured similarly, using NADH (0.2 mM in PBS, pH 7.4) and 1.5 IU LDH per mL; 20 µL samples were incubated with 180 µL reaction buffer (0.9 IU LDH/well), and absorbance was recorded at 340 nm after 15 min. Lactate and pyruvate concentrations were calculated from standard curves generated using known concentrations of sodium L-lactate and sodium pyruvate standards. A standard curve was prepared using lactate and pyruvate standards (1.6–0.025 mM) in a 96-well plate. Optical density at 570 nm was measured, and sample concentrations were determined by interpolation from the standard curve.

### Quantification and statistical analysis

Data are presented as mean ± SEM. Statistical analysis was performed using GraphPad Prism 10. Normality of data distribution was assessed using the Shapiro–Wilk test. For non-normally distributed data (e.g., microbead size), the Mann–Whitney U test was applied. For parametric data, one-way or two-way ANOVA was used as appropriate, followed by Tukey’s multiple comparisons test. Significance was defined as: ns (*p* > 0.05), *p ≤ 0.05, **p ≤ 0.01, ***p ≤ 0.001, ****p ≤ 0.00001. Sample size selection was guided by a power calculation for paired analyses, assuming a standard deviation not exceeding 20% of the mean, indicating that *n* = 3 would be sufficient to detect differences of approximately 30% between group means.

## Results

### Characterisation, viability, and actin network morphology of MSCs encapsulated in clinical-grade alginate

**Fig. 1:**
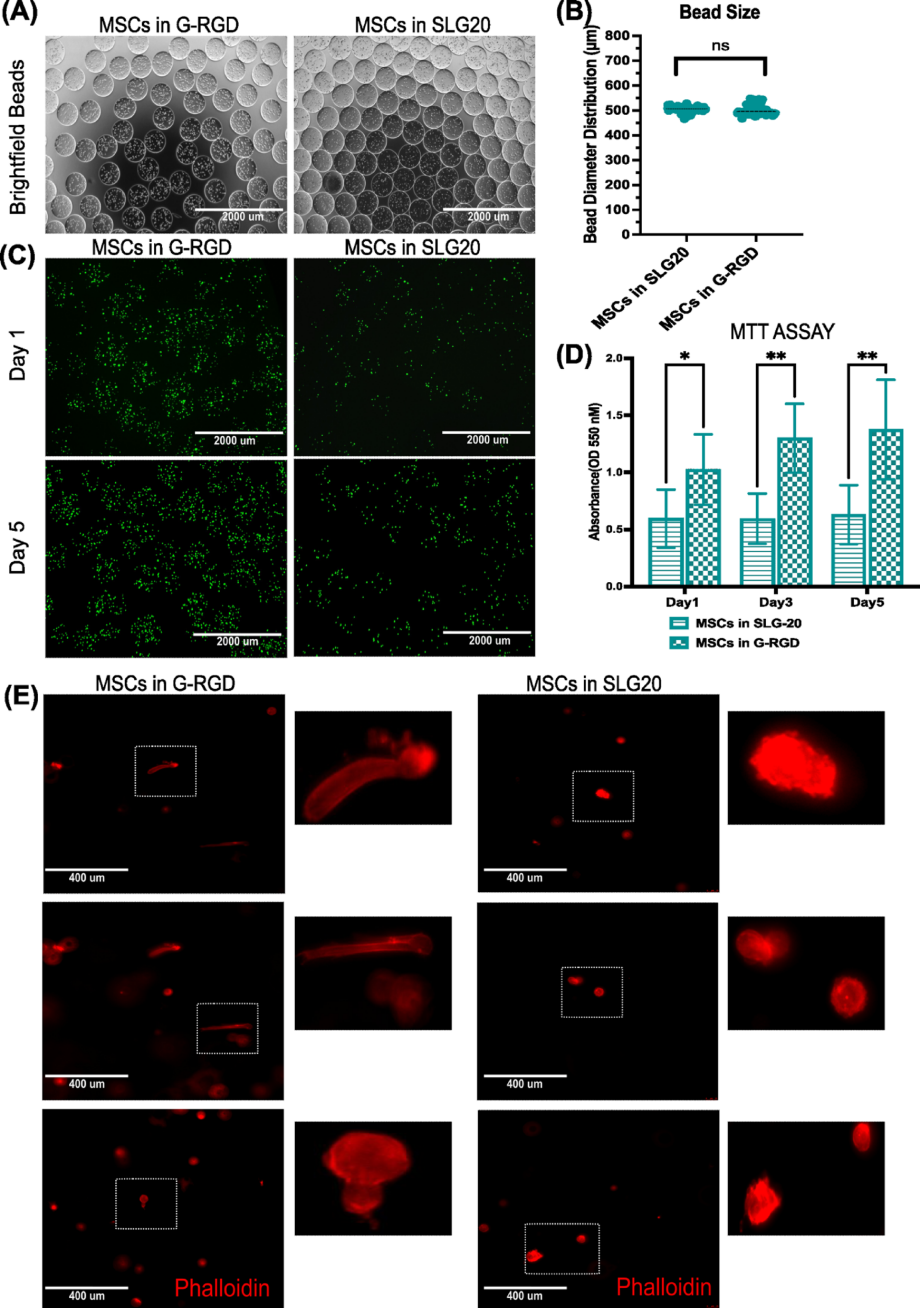
Viability, metabolic activity, and morphology of post-thawed MSCs encapsulated in clinical-grade alginates. (**A**) Following microbead fabrication, bright-field images were taken on day 0 using the EVOS™ FL Auto Imaging System (Thermo Fisher Scientific). Both unmodified SLG20 and modified G-RGD alginate produced morphologically uniform, well-defined spherical beads. Scale bar: 2000 μm. (**B**) Quantification of microbead diameters using FIJI software (https://fiji.sc/). The violin plot shows bead size distribution for SLG20 and G-RGD groups (*n* = 66 beads per group, *n* = 3). Mean bead size is indicated. (**C**) FDA staining of encapsulated MSCs in SLG20 and G-RGD alginate beads on days 1 and 5. Cell viability was assessed by fluorescence imaging. Bright-field and fluorescence images were captured using the EVOS™ FL Auto Imaging System. Scale bar: 2000 μm. (**D**) MTT assay. MSCs encapsulated in SLG20 and G-RGD alginate beads were tested at specific time points over a 5-day culture period. Data are presented as mean ± SEM (*n* = 3). (**E**) Fluorescence imaging of the actin network morphology in MSCs encapsulated in SLG20 and G-RGD alginate beads using phalloidin staining. Images were acquired using a Leica live-cell fluorescence microscope with a 20x objective lens. Brightness and contrast were adjusted using Fiji. (*n* = 3). The white rectangular box indicates the region shown in the inset, presenting a magnified view of cell morphology within the microbeads.

To investigate the impact of alginate formulations on MSCs, cells were encapsulated in either unmodified SLG20 or RGD-modified alginate (G-RGD) and cultured for up to five days. We used low-viscosity ultrapure sodium alginate with a guluronate (G) content above 60%, specifically the PRONOVA™ SLG20 or NOVATACH™ MVG GRGDSP formulations from NovaMatrix. As shown in Fig. 1A, both formulations produced morphologically clear, uniform, and well-defined spherical microbeads.

The diameters of SLG20 and G-RGD microcapsules ranged between 431 and 673 μm. The mean diameter of SLG20 microcapsules was 504.34 ± 1.45 μm, while G-RGD microcapsules exhibited a mean diameter of 500.71 ± 2.02 μm (mean ± SEM), *n* = 66 beads per group from 3 independent experiments. No statistically significant difference was observed between the groups. Figure 1B shows violin plots illustrating the size distribution and mean diameter of microbeads for each alginate formulation, highlighting the tight size distribution and consistency across different compositions.

To assess the metabolic activity and cell viability of encapsulated MSCs, fluorescein diacetate (FDA) staining and an MTT assay were performed at the indicated time points. Cell viability was evaluated using FDA staining on days 1 and 5. As illustrated in Fig. 1C, G-RGD alginate enhanced cell viability and significantly increased oxidative function over time compared to SLG20 within 24 h of production (Fig. 1D).

The RGD peptide present in the G-RGD alginates serves as an anchoring motif for the encapsulated cells, which are absent in the unmodified SLG20 alginate. To gain further insight into the cell-matrix interactions and the morphology changes of encapsulated cells, a phalloidin staining was carried out to visualise the cellular actin network morphology (Fig. 1E). Within SLG20 beads, cells predominantly exhibited a rounded morphology with minimal cytoskeletal development, suggesting limited interaction with the surrounding alginate matrix. In contrast, some MSCs encapsulated in G-RGD alginate exhibited cytoplasmic spreading, with evidence of actin network morphology. This was distinct from the more diffuse and uniform cortical F-actin staining observed in SLG20 encapsulated cells. While the current image resolution does not visualise distinct stress fibres or filopodia-like protrusions, the observed cytoskeletal pattern and cell elongation suggest enhanced integrin-mediated adhesion and interaction with the RGD-modified matrix. Although some cells maintained a rounded morphology, the overall cellular architecture suggested integrin-mediated adhesion and enhanced interaction with the RGD-modified matrix. The morphology of the cells, however, was entirely different from the classical morphology of MSCs when they were cultured on 2D conventional tissue culture plastic vessels (not shown). There were also no visible direct contact cell-to-cell interactions within the microbeads.

### Encapsulation reduces OXPHOS and induces HIF1A upregulation

**Fig. 2:**
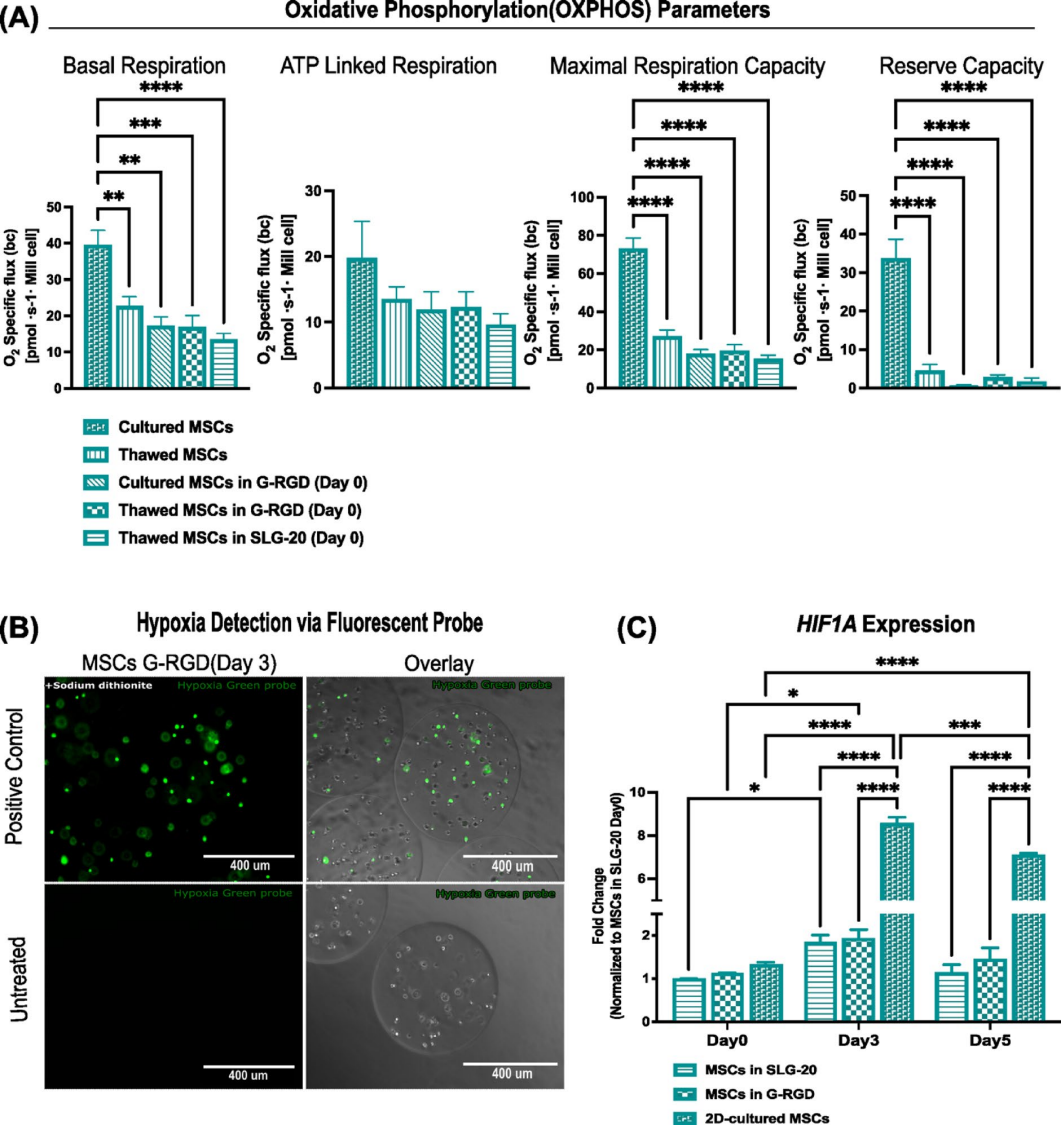
OXPHOS activity, oxygen availability, and *HIF1A* gene expression in MSCs under various encapsulation conditions. (**A**) OXPHOS Parameters, including basal respiration, ATP-linked respiration, maximal respiratory capacity, and reserve capacity, were assessed using Oroboros high-resolution respirometry. Analyses were conducted on passage 4 MSCs maintained in 2D culture (*n* = 6), cryopreserved-thawed MSCs (*n* = 9), and MSCs that were either thawed and encapsulated in SLG20 or G-RGD alginate (*n* = 3 per group), or cultured and subsequently encapsulated in G-RGD alginate (*n* = 4), all assessed immediately after encapsulation (Day 0). Cells or alginate microbeads were sequentially exposed to defined substrates and inhibitors to interrogate distinct components of the mitochondrial respiratory chain. OXPHOS were recorded in real-time and normalised to cell number, expressed as pmol O₂·s⁻¹·10⁻⁶ cells (specific flux). (**B**) Microbeads were stained with 5 µM Image-iT™ Green Hypoxia Reagent (Invitrogen™) and incubated for 4 h. Sodium dithionite-treated beads (top left) showed strong fluorescence, confirming probe sensitivity under hypoxic conditions. Minimal fluorescence signal was observed in MSCs encapsulated in G-RGD alginate at all time points (up to Day 5); representative images from Day 3 microbeads are shown here. Images were acquired using an inverted fluorescence microscope. Scale bars = 2000 μm. (**C**) *HIF1A* mRNA was assessed via qRT-PCR in encapsulated MSCs over time (*n* = 3). Fold changes in MSCs encapsulated in SLG20 versus G-RGD, normalised to SLG20 Day 0. Comparison including 2D-cultured MSCs, highlighting time-dependent upregulation of *HIF1A* expression in 3D-encapsulated conditions. Statistical analysis by one-way ANOVA with Tukey’s post hoc test; data are presented as mean ± SEM from at least three independent experiments (*n* ≥ 3). *p < 0.05, **p < 0.01, ***p < 0.001, and ****p < 0.0001; ns, not significant (GraphPad Prism10).

To investigate how encapsulation and alginate composition influence MSC bioenergetics, mitochondrial function was assessed by high-resolution respirometry using an Oroboros instrument (Fig. 2A). Basal respiration, assessed in William’s E medium supplemented with 10% FBS, was significantly higher in 2D-cultured MSCs (40.0 ± 4.5 pmol O₂·s⁻¹·10⁻⁶ cells) than in thawed MSCs (23.4 ± 3.0 pmol O₂·s⁻¹·10⁻⁶ cells). The encapsulation resulted in a 58% decrease oxygen consumption rate as cultured MSCs encapsulated in G-RGD showed a respiration of 17 ± 2.0pmol O₂·s⁻¹·10⁻⁶ cells (*p* < 0.01). The encapsulation of cryopreserved/thawed MSCs in G-RGD, or in SLG20 did not result in significant changes in respiration (14.9 ± 1.6 or 12.4 ± 1.5 pmol O₂·s⁻¹·10⁻⁶ cells, respectively, ns). Similarly, the maximal respiratory capacity was highest in 2D-cultured MSCs (73.2 ± 5.4 pmol O₂·s⁻¹·10⁻⁶ cells) as compared to all thawed and encapsulated groups, with the lowest values observed in SLG20-encapsulated MSCs (15.2 ± 2.2 pmol O₂·s⁻¹·10⁻⁶ cells; *p* < 0.0001). Notably, no significant differences emerged between SLG20 and G-RGD encapsulation conditions in any of the OXPHOS parameters assessed.

ATP-linked respiration and proton leak-linked OCR seemed also to be reduced following MSC encapsulation, although these differences did not achieve statistical significance (Fig. 2A). As expected, thawed MSCs displayed reduced basal and maximal respiration compared to cultured MSCs, indicating impaired post-thaw mitochondrial function. While the encapsulation of thawed MSCs tended to further decrease OXPHOS activity relative to 2D culture, no significant differences were observed between thawed and encapsulated cells at day 0, nor between the two alginate formulations. To analyse the effects of encapsulation in the recapitulation of the physiological MSC niche, we next studied oxygen availability in alginate, using an oxygen-sensitive fluorescent probe (Fig. 2B). As a positive control, sodium dithionite was used to scavenge oxygen from the beads. These beads showed a strong fluorescence signal, confirming the probe responsiveness under oxygen-depleted conditions. However, a minimal signal was observed in G-RGD encapsulated MSCs at day 3, seemingly indicating no substantial hypoxia or diffusion barrier within the alginate matrix.

To investigate hypoxic adaptation under 3D versus 2D culture, we next analysed expression levels of *HIF1A*, a key transcription factor regulating cellular responses to oxygen availability. Despite the lack of detectable hypoxia using the oxygen-sensitive fluorescent probe (Fig. 2B), encapsulated MSCs exhibited significant upregulation of *HIF1A* gene expression over time (Fig. 2C). *HIF1A* mRNA expression increased significantly by Day 3 in both SLG20 and G-RGD encapsulated MSCs. Specifically, levels rose 88% from 1.00 ± 0.00 on Day 0 to 1.88 ± 0.19 in SLG20 (*p* < 0.05), and 71% from 1.18 ± 0.03 to 2.02 ± 0.11 in G-RGD (*p* < 0.0001), No statistically significant differences were observed between SLG20 and G-RGD at any time point, indicating that matrix composition did not influence *HIF1A* expression under these conditions. Unexpectedly, 2D-cultured MSCs showed much higher *HIF1A* expression, reaching 8.57 ± 0.47 at Day 3 and 7.10 ± 0.10 at Day 5 (*p* < 0.0001 vs. both encapsulated groups), suggesting a distinct transcriptional response to monolayer culture.

### Time-dependent OXPHOS and metabolic shift in encapsulated MSCs

**Fig. 3:**
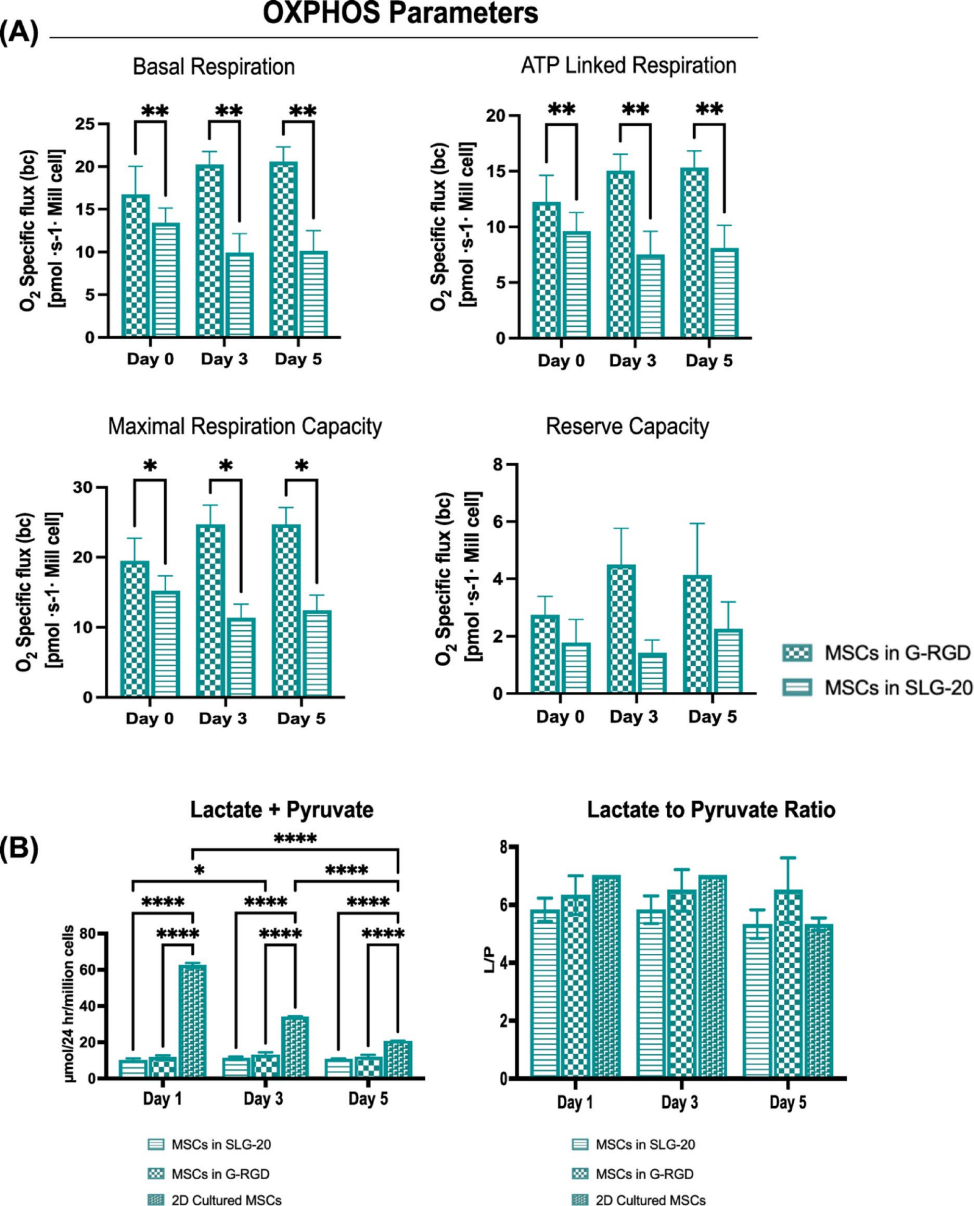
Time-Dependent OXPHOS Dynamics and Glycolytic output in Encapsulated MSCs (**A**) OXPHOS—including basal, ATP-linked, maximal, and reserve capacity—was assessed in thawed MSCs encapsulated in G-RGD or SLG20 alginate at day 0 and subsequent time points using Oroboros high-resolution respirometry. Microbeads were exposed to defined mitochondrial substrates and inhibitors, and oxygen consumption was measured in real-time, normalised to cell number and expressed as pmol O₂·s⁻¹·10⁻⁶ cells (specific flux). (**B**) Total extracellular lactate + pyruvate levels and lactate-to-pyruvate ratio (L/P) were measured on days 1, 3, and 5 in MSCs encapsulated in SLG-20, G-RGD, and cultured in 2D. (**A**, **B**) Data are shown as mean ± SEM (*n* = 3). Statistical analysis used two-way ANOVA with Tukey’s post hoc test. Significance levels are indicated as follows: *p < 0.05, **p < 0.01, ***p < 0.001, ****p < 0.0001; ns = not significant.

To assess the impact of prolonged encapsulation on OXPHOS and metabolic activity, we evaluated the respiratory profile of thawed MSCs encapsulated in SLG20 versus G-RGD alginate over five days. Basal respiration remained consistently higher in MSCs encapsulated in G-RGD compared to SLG20 across all time points (days 0, 3, and 5; *p* < 0.01; Fig. 3A). ATP-linked respiration, representing oxygen consumption coupled to ATP synthesis, exhibited a similar pattern, with significantly greater values observed in the G-RGD group at each time point (*p* < 0.01), suggesting enhanced mitochondrial OXPHOS coupling within the RGD-functionalised matrix. Maximal respiratory capacity, defined as peak oxygen consumption under uncoupled conditions, was also significantly elevated in G-RGD encapsulated MSCs (*p* < 0.05 at all time points), indicating an improved ability to meet increased energetic demands. As a result, the reserve respiratory capacity, calculated as the difference between maximal and basal respiration, was increased, but did not reach a significant difference between the two groups.

To further characterise the metabolic state of MSCs within alginate, lactate and pyruvate levels were measured as indicators of glycolytic activity and the cytosolic redox potential, a marker of mitochondrial function (Fig. 3B). Metabolic analysis revealed a strong glycolysis (lactate + pyruvate levels) in 2D-cultured MSCs compared to encapsulated cells from day 1, which decreased over time (from 62.3 ± 1.5 to 20.7 ± 0.2, respectively). In contrast, encapsulated MSCs in both SLG-20 and G-RGD hydrogels secreted lower levels of lactate and pyruvate (*p* < 0.0001 for all comparisons). Interestingly, the lactate-to-pyruvate (L/P) ratio remained relatively stable across groups, indicating that despite differences in total flux, the cellular redox balance (NADH /NAD^+^) was maintained.

### Alginate-mediated regulation of mitochondrial biogenesis

**Fig. 4:**
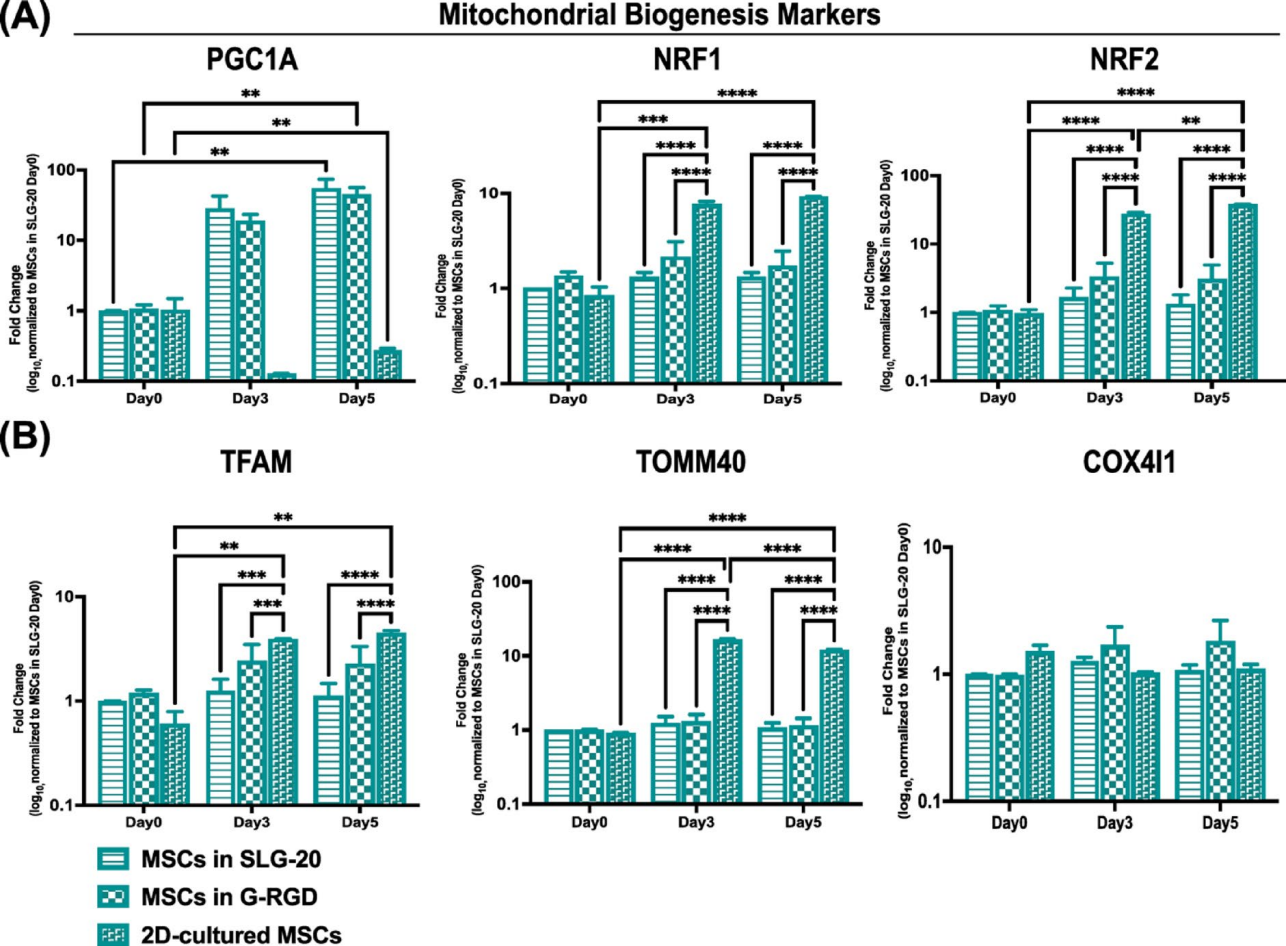
Gene expression analysis of mitochondrial biogenesis and function in MSCs encapsulated in alginate hydrogels. (**A**) Quantitative RT-PCR analysis of mitochondrial biogenesis-associated genes *PGC1A*, *NRF1*, and *NRF2* in MSCs encapsulated in SLG20 or G-RGD alginate hydrogels at days 0, 3, and 5. MSCs cultured in conventional 2D conditions were included as controls. Gene expression was normalised to the mean of *GAPDH*, *ACTB*, and *B2M* housekeeping genes. All expression values were log₁₀-transformed and normalised to SLG20 Day 0 to enable comparative fold-change analysis. (**B**) Expression of genes involved in mitochondrial structure and respiratory function, *TFAM*, *TOMM40*, and *COX4I1*, was assessed in parallel under the same experimental conditions. (**A**, **B**) Statistical analysis was performed using two-way ANOVA followed by Tukey’s multiple comparisons test. *p < 0.05, **p < 0.01, ***p < 0.001, ****p < 0.0001.

Because of the effects measured above of the cell encapsulation on cell metabolism and OXPHOS, we next wanted to investigate the long-term impact of 3D encapsulation on mitochondrial biogenesis pathways; we analysed the expression of key regulatory genes in MSCs encapsulated in SLG20 or G-RGD alginate at days 0, 3, and 5, with 2D-cultured MSCs included as a reference. *PGC1A*, a master regulator of mitochondrial biogenesis, was significantly upregulated over time in both hydrogel groups compared to 2D culture, respectively 60 and 40-fold on day 5 as compared to day 0 (Fig. 4A), whilst its expression decreased in cells cultured in conventional 2D-culture plates (reduced 66% on day 5 as compared to day 0).

Despite the strong upregulation of *PGC1A* mRNA in MSCs encapsulated in both SLG-20 and G-RGD alginate, no significant time-dependent changes were observed in downstream mitochondrial biogenesis regulators such as *NRF1*,* NRF2*, or *TFAM*. Similarly, the expression of genes encoding outer or inner mitochondrial membrane proteins, including *TOMM40*, remained stable over time within encapsulated conditions. In contrast, 2D-cultured MSCs showed a marked increase in *NRF1*, *NRF2*, *TFAM*, and *TOMM40* expression at days 3 and 5 compared to day 0. Interestingly, *COX4I1* expression remained unchanged across all conditions and time points (Fig. 4B).

Collectively, these data indicate that while mitochondrial biogenesis is transcriptionally activated in 3D hydrogels, the extent and kinetics differ markedly from 2D culture.

**Fig. 5:**
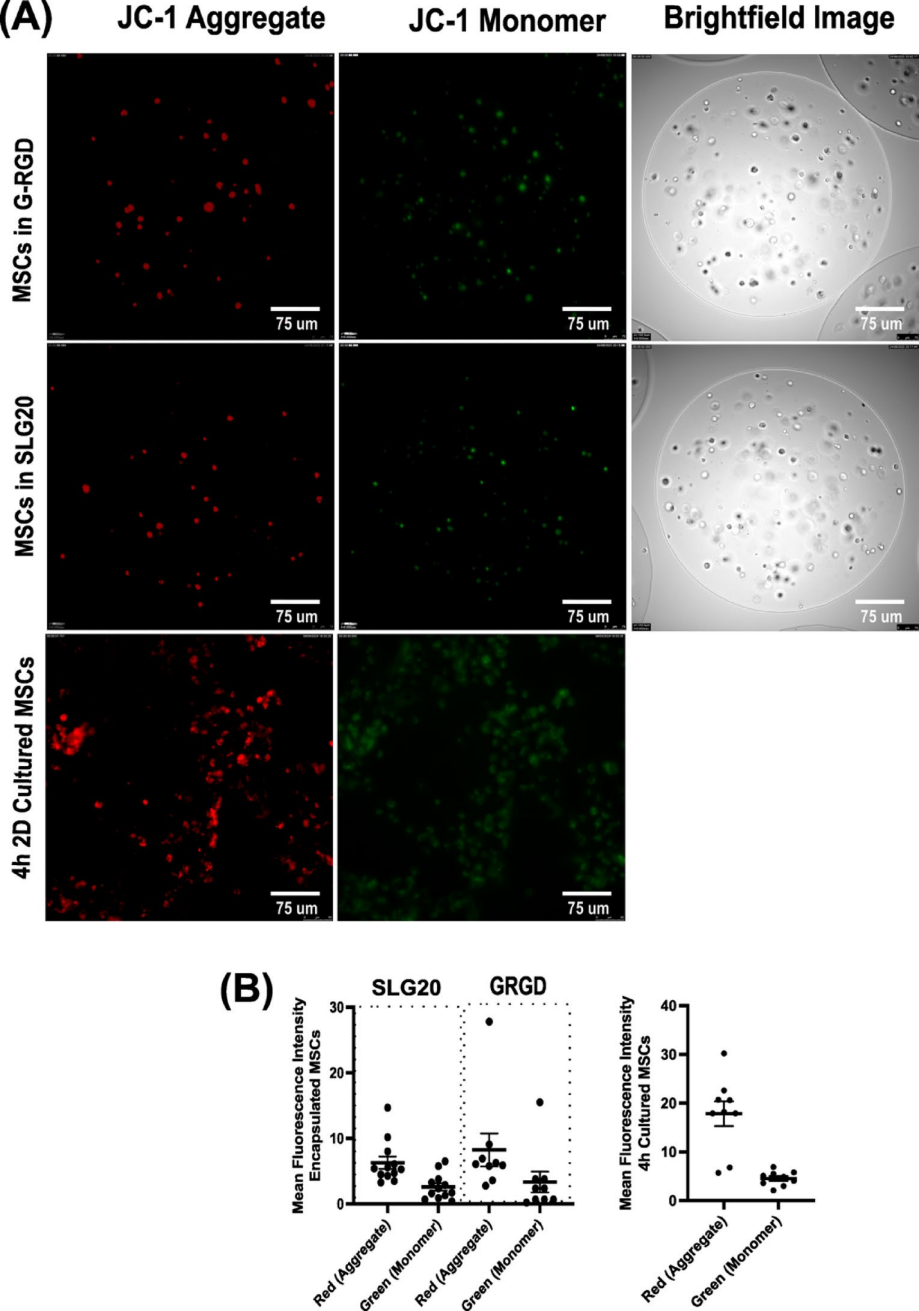
Assessment of mitochondrial membrane potential in encapsulated MSCs and 2D MSCs using JC-1 staining. (**A**) Representative fluorescence microscopy images of MSCs encapsulated in SLG20 or G-RGD alginate hydrogels. Red fluorescence indicates JC-1 aggregates, reflecting polarised mitochondria with high membrane potential, while green fluorescence indicates JC-1 monomers associated with mitochondrial depolarisation. and corresponding brightfield images were included to visualise bead morphology (2D cultured not shown). (**B**) Mitochondrial membrane potential was quantified by calculating the red-to-green fluorescence intensity ratio using Fiji (ImageJ). Data represent the mean ± SEM from 8–10 microbeads per experiment across three independent experiments.

### Reduced mitochondrial membrane potential in encapsulated MSCs

To further evaluate mitochondrial activity, JC-1 staining was employed to assess the mitochondrial membrane potential (ΔΨm) in encapsulated MSCs. The cationic dye JC-1 accumulates in mitochondria and aggregates as a function of the ΔΨm, emitting red fluorescence, whereas green-fluorescing monomers reflect depolarised mitochondria.

As shown in Fig. 5A, in encapsulated MSCs, both SLG20 and G-RGD groups showed most cells with red fluorescence and some green signal, indicative of some lower mitochondrial polarisation. In contrast, 2D-cultured MSCs exhibited a markedly elevated red JC-1 fluorescence with minimal green signal, suggesting a higher ΔΨm and overall mitochondrial activity, which correlates with the OXPHOS data. Quantitative analysis of mean fluorescence intensity (Fig. 5B) confirmed that red fluorescence was significantly higher in 2D MSCs compared to either encapsulated group. Among encapsulated MSCs, the G-RGD group showed slightly higher red intensity than SLG20.

**Fig. 6:**
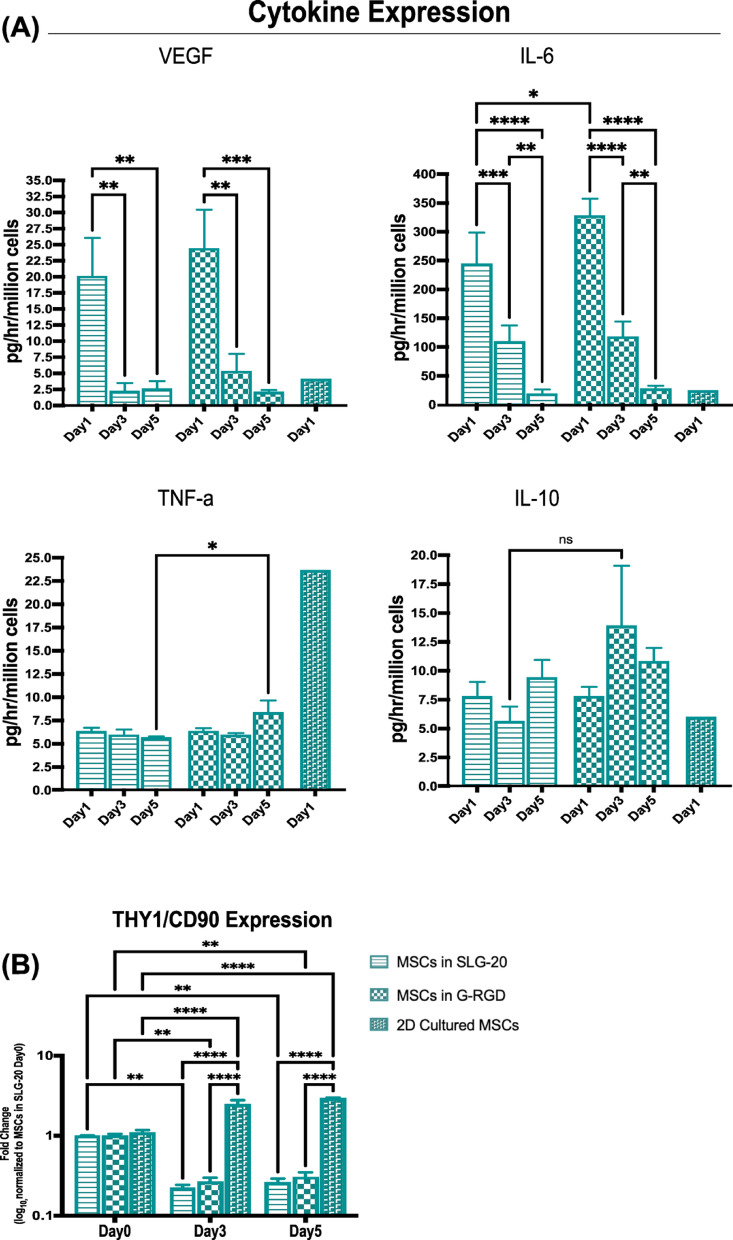
Time-Dependent Cytokine Secretion and *THY1/(CD90)* expression in encapsulated MSCs. (**A**) Quantification of VEGF, IL-6, TNF-α, and IL-10 levels in culture supernatants collected from MSCs encapsulated in SLG20 or G-RGD alginate microbeads over time. 2D-cultured MSCs were included only at Day 1 as a baseline reference for early cytokine release before encapsulation-induced adaptation. Cytokine concentrations are expressed as pg/hr/million cells. (**B**) *THY1*(*CD90*) gene expression was evaluated via qPCR and normalised to SLG-20 Day 0. Data are shown as mean ± SEM, *p < 0.05, **p < 0.01, ***p < 0.001, ****p < 0.0001.

### Enhanced cytokine secretion and altered stemness marker expression in encapsulated MSCs

Given that *HIF1A* regulates VEGF expression, we next quantified VEGF secretion from MSCs following encapsulation. Conventional 2D-cultured MSCs were included as a Day 1 control to reflect baseline paracrine output prior to encapsulation-induced adaptation; however, statistical comparisons between 2D and encapsulated groups were not performed due to limited sample size (*n* = 1 for 2D). As illustrated in Fig. 6A, at day 1, encapsulated MSCs showed a strong VEGF production, which is then rapidly downregulated from day 3, for both types of alginates. Interestingly, whilst *HIF1A* was upregulated in 2D-cultured cells, their VEGF expression was low here, at day 1. While a trend toward higher VEGF secretion in G-RGD compared to SLG20 was observed at Day 3, no statistically significant differences between the two alginate groups were detected at any time point.

To further characterise the immunomodulatory secretome of MSCs, we next measured additional cytokines including IL-6, TNF-α, and IL-10. IL-6 levels, which were low in 2D-cultured cells, increased dramatically in encapsulated MSCs. In G-RGD alginate, the cell secretion of IL-6 reached significantly 34.5% higher levels than in SLG-20, on day 1 (327.9 vs. 243.8 pg/hr/million cells, *p* < 0.05). For both types of alginates, the secretion then strongly decreased, reaching 8.3% and 7.7% of their initial values, respectively for G-RGD and SLG20.

Although TNF-α secretion remained low overall, G-RGD encapsulated MSCs exhibited a statistically different 49% increase over SLG20 by day 5 (*p* < 0.05). In contrast, 2D-cultured MSCs exhibited higher TNF-α expression than when encapsulated. While no statistically significant differences in IL-10 levels were observed between the groups, as represented in Fig. 6A, G-RGD-encapsulated MSCs seemed to display slightly higher levels compared to SLG-20.

To examine stemness-related changes, *THY1(CD90)* gene expression was evaluated over five days (Fig. 6B). While 2D MSCs showed a steady increase in *THY1* expression over time (1 vs. 2 on day 1 vs. day 5; *p* < 0.0001), both SLG20 and G-RGD encapsulated MSCs displayed significantly reduced levels throughout the culture period, with no significant differences between day 3 and day 5.

**Fig. 7:**
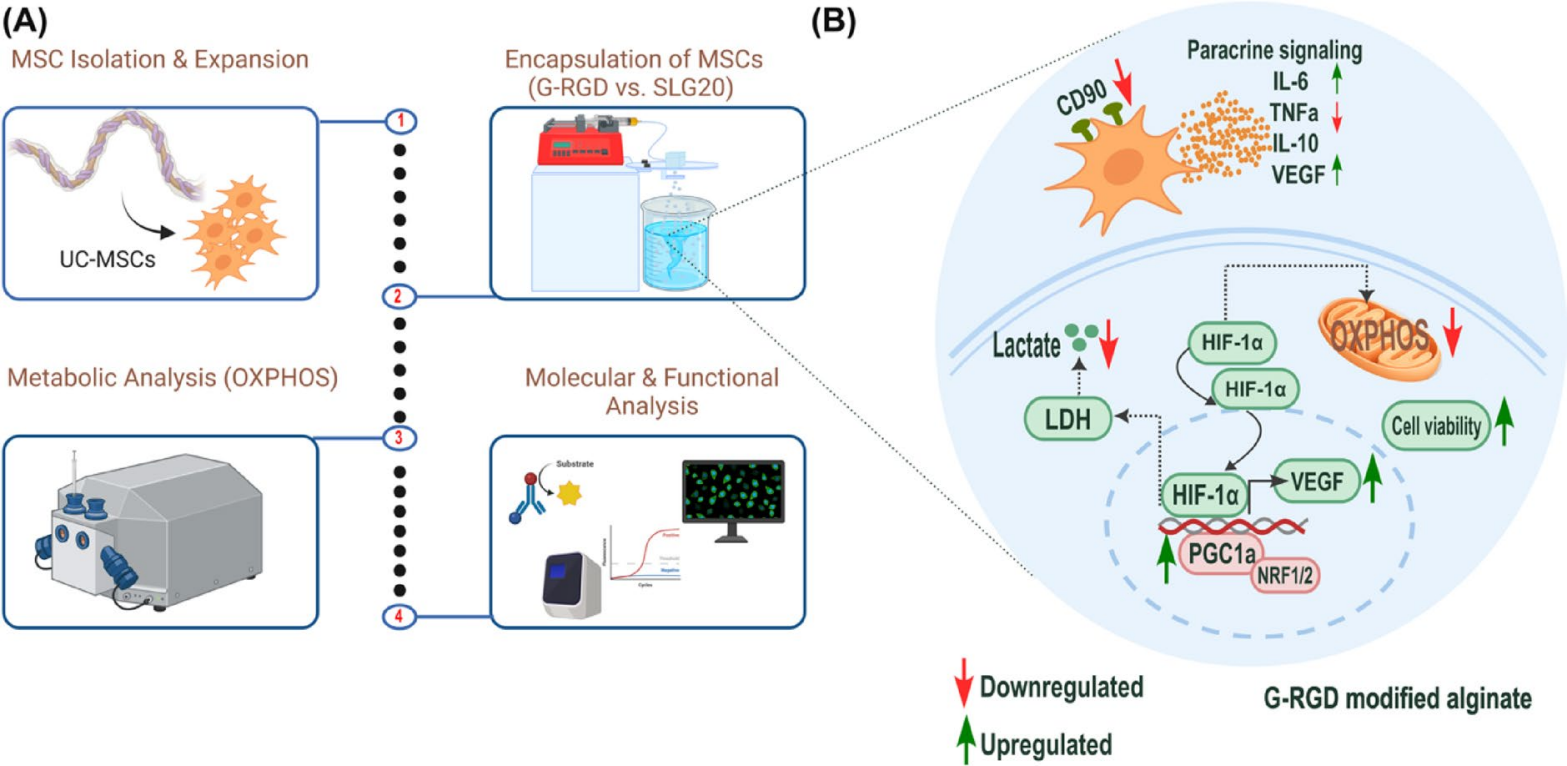
Schematic overview of the experimental study design and key functional outcomes associated with G-RGD modified alginate. (**A**) Overview of the study workflow: (1) isolation and expansion of umbilical cord-derived mesenchymal stromal cells (UC-MSCs); (2) encapsulation of MSCs in GMP-compliant alginate formulations (G-RGD vs. SLG20), following standard expansion and cryopreservation workflows as described in Methods. (3) metabolic analysis of mitochondrial function (OXPHOS); and (4) molecular and functional assays. (**B**) Summary of major findings in G-RGD-encapsulated MSCs, including modulation of HIF-1α signalling, VEGF, cytokine secretion, lactate production, OXPHOS, and cell viability. Upward and downward arrows indicate upregulation and downregulation, respectively. All assays were performed in vitro using *N* = 3 biological replicates.

Schematic representation of the experimental workflow, encapsulation procedure, and major functional outcomes associated with G-RGD–modified alginate, as illustrated in Fig. 7.

## Discussion

This study aimed to investigate how alginate encapsulation, with or without peptide-modification, modulates UC-MSC viability, mitochondrial function, and cytokine secretion, with a focus on time-dependent metabolic and transcriptional adaptations. We focused on the comparison of two GMP-grade alginate matrices (SLG20 vs. G-RGD) for encapsulation of cryopreserved UC-MSCs, and analysed their impact on the cell bioenergetics, metabolic profiling, and baseline paracrine secretion. Our findings refine the existing RGD–alginate literature by evaluating clinically relevant, FDA and MHRA-controlled materials and workflows, defining how matrix bio functionality shapes MSC metabolic and secretory adaptation over time.

One of the most compelling and unexpected findings was that, although G-RGD encapsulation improved overall metabolic function and cell viability, it led to a significant suppression of the cell oxygen consumption rate, including their maximal oxygen consumption capacity obtained when the OXPHOS is uncoupled with CCCP. This happened on day 0, from the moment the cells were encapsulated. The maximal oxygen consumption capacity for normal isolated cells is usually directly controlled by the overall mass of respiratory chain in the cells and remains steady until there is no energy substrate to fuel the OXPHOS, or no oxygen. As the measurement was started a few minutes after the cell encapsulation, the change in uncoupled respiration cannot be due to a downregulation of their protein expression. Taken together, the immediate reduction in OCR following encapsulation is consistent with a rapid shift in cellular bioenergetics induced by the 3D environment. Though we did not directly quantify the oxygen tension inside the beads, and an oxygen-sensitive dye did not indicate any overt hypoxia, the fact that the MSCs had an increased HIF1A mRNA expression strongly supports the notion that the encapsulation process resulted in a hypoxic niche for the cells. The much higher *HIF1A* expression observed in 2D MSCs was unexpected, but this finding is consistent with previous work showing (1) that MSC HIF1a expression increases with cell density^28^, which happened only in the 2D-cultured cells and (2) that MSCs derived from paediatric bone marrow or umbilical-cord blood further stabilise *HIF1A* under normoxia via upregulated transcription^29^. However, in the light of what was measured in 2D cells, the level of *HIF1A* mRNA increase in encapsulated cells was only a moderate one.

HIF-1α protein expression acute regulation does not depend on mRNA expression level but on its stabilisation during hypoxia, as it is constitutively targeted for ubiquitin-proteasome degradation in conditions of normoxia. Hence, an analysis of HIF1A protein by western blot or immunofluorescence would have confirmed the immediate hypoxia-sensing capacity of our cells once encapsulated. We did not measure HIF-1α protein stabilisation in this study; future work should therefore include the quantification of (1) intra-bead oxygen tension and (2) HIF-1α protein (e.g., IF/Western blot) alongside any pathway modification. Besides its regulation by the oxygen tension, *HIF1A* has been shown to be affected by the mechanotransduction properties of their extracellular matrix^30^, so the changes in *HIF1A* in our encapsulated cells could also stem from the inherent alginate properties. These findings suggest that matrix biofunctionality may influence hypoxia‑associated signalling, although this remains speculative in the absence of direct pathway analysis. Encapsulation was associated with a distinct bioenergetic phenotype, consistent with broader effects of 3D biophysical environments reported in the literature^31,32^.

In line with this altered bioenergetic profile, encapsulated MSCs showed upregulation of *PGC1A*, a key regulator of mitochondrial biogenesis. However, in the 5-day timeframe of sample collection, *NRF1* and *TFAM* did not show any statistically significant changes. This stability, combined with reduced OXPHOS activity and the absence of a compensatory increase in glycolysis, indicates an overall slowdown of metabolic activity in encapsulated MSCs. The decrease in OXPHOS activity when the cells are encapsulated, together with a low mitochondrial membrane potential, a constant cytosolic redox potential and low glycolysis, points to an overall decrease in cell activity^18,33^.

Among encapsulated cells, the G-RGD-encapsulated MSCs showed higher viability, improved mitochondrial respiratory parameters over time, and distinct actin network morphology compared to SLG20. These findings are consistent with previous reports that encapsulation within biofunctional hydrogels enhances MSC viability and metabolic stability^11,34^. Integrin engagement with RGD peptides triggers cytoskeletal signalling and survival pathways. In our study, phalloidin staining showed some cellular morphology changes in G-RGD encapsulated cells, totally absent in SLG20 alginate, suggesting a specific cell-matrix interaction that may explain some of the functional improvements seen in the cells^1,2,8^. From a biomaterial standpoint, sodium alginate is attractive for clinical use, especially for short-term applications requiring non-vascularised advanced therapy medicinal products, due to its biocompatibility, ease of cross-linking with divalent cations, and GMP scalability^7,35^. However, its lack of native cell adhesion sites makes it biologically inert, preventing integrin-mediated signalling^13^. RGD functionalisation addresses this by introducing specific binding motifs that enhance cytoskeletal organisation, survival signalling, and paracrine function^34,36^. Components of the extracellular matrices, such as fibrin, collagen, hyaluronic acid, or complex decellularized ECM hydrogels, would provide richer biochemical cues but often present challenges in standardisation, immunogenicity, and large-scale manufacturing compared to alginate^13^.

The function of cells is not solely influenced by their capacity to adhere to their matrix. The permeability and stiffness of the matrix also strongly impacts MSC phenotype^37,38^. Preliminary optimisation experiments of our work indicated differences in stiffness between the SLG20 and G-RGD alginates, which led to the use of a lower alginate concentration for G-RGD beads; however, rheological measurements were not performed, therefore any resulting stiffness-related effects on MSC behaviour cannot be excluded. Low levels of RGD substitution have been reported not to substantially alter the bulk stiffness or mesh size of alginate hydrogels^39,40^. Future work will include rheological characterisation to confirm any differences between SLG20 and G-RGD.

MSCs are widely utilised for their immunomodulatory and paracrine functions, often requiring pre-licensing with inflammatory stimuli to trigger a cytokine release relevant for their specific therapeutic applications^41^. In this study, cytokine secretion was assessed under baseline, unstimulated conditions without prior inflammatory priming (‘licensing’), providing insight into the intrinsic paracrine activity of encapsulated MSCs. We observed that VEGF and IL-6 levels were higher in encapsulated MSCs at Day 1 compared to 2D-cultured MSCs, with the 2D Day 1 condition included as a descriptive baseline reference only (*n* = 1) and not used for statistical comparisons. No significant differences in VEGF secretion were detected between SLG20 and G-RGD encapsulated MSCs at any time point. In contrast, IL-6 secretion was modestly but significantly higher in G-RGD compared to SLG20 at early time points, suggesting a potential contribution of matrix biofunctionality to early paracrine modulation^42,43^. However, both VEGF and IL-6 secretion declined markedly over time in both encapsulated cell groups, suggesting an initial burst of cytokine release that was not sustained in the absence of continued inflammatory stimuli. This may indicate that while the encapsulation microenvironment can transiently enhance MSC paracrine output, ongoing stimulation may be necessary to maintain cytokine secretion relevant for prolonged therapeutic efficacy^44–46^. Interestingly, TNF-α levels remained low overall, as compared to 2D cultures, for all time points studied, whilst IL-10 secretion was modestly elevated in encapsulated groups, suggesting maintained or improved immunosuppressive potential^5,47^. Overall, this points to a change in the cell functional priorities when encapsulated, with further modulations when their encapsulation matrix is bio-mimetic. These findings align with previous studies showing that alginate encapsulation can support baseline cytokine secretion under non-inflammatory conditions, without implying sustained or long-term effects^48,49^.

To briefly verify the impact of encapsulation on one marker of MSC identity, we analysed *THY1(CD90)* expression. It was significantly reduced in encapsulated MSCs, in both groups, from day 3. This finding is consistent with previous reports of downregulated surface markers in non-adherent 3D environments and may reflect a shift in cell identity^41,50^. In one of our other work (unpublished) transcriptomic analysis of MSCs in G-RGD alginate (day 0 vs. day 5) showed upregulation of *CD105* and downregulation of *KLF4*, while other identity and stemness markers remained largely unchanged, so the extent of phenotype change is unclear at this stage. Functional assays such as trilineage differentiation, CFU-F, or proliferation studies, not undertaken here, would have been more conclusive to determine whether encapsulation alters MSC stemness. Future studies, including full cell identity analysis, will be important to validate these phenotypic observations.

From a translational perspective, our study is particularly relevant to producing MSC microbeads and the design of off-the-shelf cell therapies using GMP-compliant MSCs. As our encapsulation experiments were conducted using cryopreserved and thawed MSCs, these findings directly inform workflows requiring rapid deployment of pre-tested, cryopreserved clinical cell sources while maintaining functional performance^51,52^. The ~ 500 μm microbead diameter used in our study was selected based on previous findings that this size offers a favourable balance between oxygen, nutrient diffusion, the maintenance of cell viability and capsule integrity^53,54^. Our in vitro data will inform formulation selection and assay prioritisation for subsequent translational studies (e.g., co-encapsulation workflows), rather than demonstrating therapeutic efficacy^59^.

Our study was limited in time, but the current results warrant further investigation. Although PGC1A expression similarly increased over time in SLG20 and G-RGD groups, the greater OXPHOS activity and early cytokine output in the G-RGD alginate suggest a potential additive effect of RGD–integrin signalling on top of general 3D-induced metabolic adaptations. While we hypothesise that RGD engagement triggers FAK/PI3K/Akt pathway activation to support this enhanced metabolic state, direct mechanistic confirmation is currently lacking. We acknowledge this as a study limitation and propose future work involving pathway inhibition (e.g., FAK or integrin-blocking antibodies) and phospho-protein analysis. Additionally, further in vivo validation will be essential to determine whether these metabolic advantages result in improved therapeutic efficacy using either immunocompromised or humanised animal models to verify the functional impact of G-RGD encapsulation on MSC therapeutic efficacy. Another limitation of this study is that we did not directly measure rheological or mechanical properties (e.g., storage modulus, mesh size, diffusion coefficients) of SLG20 versus G-RGD microbeads. Although RGD substitution was relatively low (< 0.5%) and crosslinking conditions were standardised, subtle differences in stiffness cannot be excluded and may contribute to MSC behaviour. Future work will incorporate rheological testing and permeability measurements alongside functional assays.

Taken together, our findings underscore the importance of matrix composition in directing MSC fate and function. This study provides a translation‑focused comparison of GMP‑grade G‑RGD and unmodified SLG20 alginate for the encapsulation of cryopreserved UC‑MSCs, integrating the assessments of mitochondrial bioenergetics and paracrine secretion profile. This translation-ready workflow, using appropriately sized (~ 500 μm) GMP-compliant microbeads optimised for intraperitoneal delivery in paediatric acute liver failure, provides in vitro evidence describing how matrix composition and 3D culture influence MSC metabolic and paracrine adaptation.

## Conclusions

Our study aimed to compare two GMP-grade SLG20 and RGD-functionalised alginate matrices for MSC encapsulation, assessing their effects on viability, metabolism, and cytokine secretion. We found that encapsulation maintained MSC viability, reduced OXPHOS and glycolysis, and transiently increased VEGF and IL-6 secretion. IL-10 levels remained unchanged over time. RGD-modified alginate further enhanced MSC viability, improved bioenergetic function, and induced changes in cell morphology. These findings highlight the potential of GMP-grade G-RGD alginate for future translation, as a sole cell component or co-encapsulated support of different therapeutic cells^59^.

## Data Availability

All data generated or analysed during this study are included in this published article. Data supporting the findings of this study are available from the corresponding author upon reasonable request.
